# Surgical Management of Breast Cancer Adjacent to a Calcified Ventriculoperitoneal Shunt: A Case Report

**DOI:** 10.70352/scrj.cr.25-0626

**Published:** 2026-02-07

**Authors:** Hitomi Wake, Katsushige Watanabe, Hideaki Tanami

**Affiliations:** 1 Department of Surgery, Tokyo Metropolitan Matsuzawa Hospital, Tokyo, Japan; 2 Department of Neurosurgery, Tokyo Metropolitan Matsuzawa Hospital, Tokyo, Japan

**Keywords:** ventriculoperitoneal shunt, breast cancer, rerouting of shunt

## Abstract

**INTRODUCTION:**

Breast cancer arising in tissue adjacent to a ventriculoperitoneal (VP) shunt is exceptionally rare, and there is little guidance on how to manage the shunt hardware during oncologic surgery in such cases.

**CASE PRESENTATION:**

The patient was a 46-year-old woman with a history of intellectual disability and hydrocephalus. She had undergone VP shunt placement via the left chest wall for hydrocephalus during childhood. Decades later, she was admitted to our hospital for the examination and treatment of left breast cancer. Imaging studies revealed a tumor in the left nipple, with the shunt catheter passing as close as 12 mm from the tumor. During radical mastectomy for breast cancer, it became clear that preserving the catheter was not feasible. Consequently, the shunt catheter was rerouted, and both the left breast and the catheter were removed as a single unit. Pathological findings of a resection specimen revealed invasive ductal carcinoma pT2N1M0, pStage IIB. Although the catheter had been positioned very closely to the tumor, no cancer progression was observed along the catheter.

**CONCLUSIONS:**

The present case is noteworthy for describing a rare case of mastectomy for breast cancer involving repositioning of an ipsilateral catheter. Included in this report is a review of past studies.

## Abbreviations

CSF
: cerebrospinal fluid
ER(+)
: estrogen receptor positive
HER2
: human epidermal growth factor receptor 2
PgR(+)
: progesterone receptor positive
VP shunt
: ventriculoperitoneal shunt

## INTRODUCTION

Ventriculoperitoneal (VP) shunting, which often traverses the breast, is the primary treatment for hydrocephalus in patients of all ages. Managing the catheter becomes a clinical challenge if breast cancer develops on the ipsilateral side. However, no consensus on this matter has yet been reached owing to the extreme rarity of such cases. Herein, we describe a unique case of breast cancer occurring adjacently to a VP shunt decades after its placement in early childhood, which was managed by rerouting the distal catheter before performing a radical mastectomy. Included in this report is a review of past studies.

## CASE PRESENTATION

Patient: A 46-year-old woman.

Chief complaint: Mass in the left nipple area.

Present illness: The patient visited a local clinic after noticing a lump in her left breast. Breast ultrasonography revealed two tumors in the left breast, one within the nipple measuring 16 mm, the other at the 3 o’clock position and measuring 12 mm. Analysis of a core needle biopsy specimen led to the diagnosis of both lesions as invasive breast carcinoma. The patient was admitted to our hospital for further evaluation and treatment.

Physical examination: An elastic, firm, 2-cm, mobile mass was palpated in the left nipple area. The distal shunt catheter was also palpable from the mastoid tip to the superior clavicular border, but the segment along the anterior chest and abdominal wall was not palpable.

Past medical history: The patient had intellectual disability and a remote history of infantile spasms. She had undergone VP shunt placement at 21 months of age for hydrocephalus of unknown etiology. The surgical records, valve model, and implantation center were unable to be ascertained, and the patient had not been followed routinely by a neurosurgeon since childhood.

Medications: The patient was receiving oral risperidone 1 mg once daily, oral sodium valproate 300 mg once daily, and oral diazepam 4 mg at bedtime.

Core needle biopsy: Invasive ductal carcinoma.

**Fig. 1:**
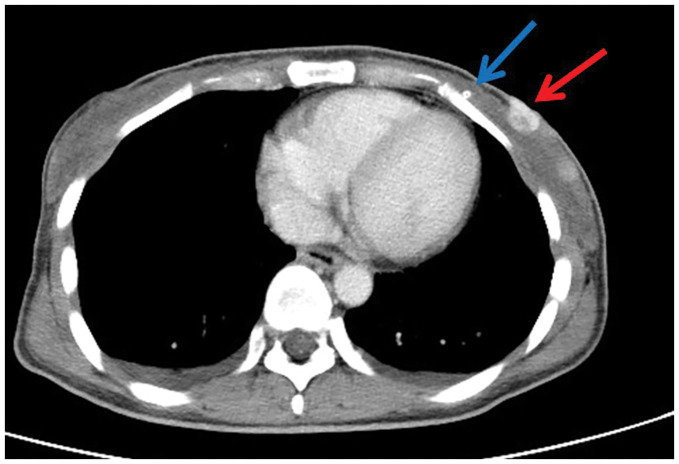
Contrast-enhanced CT of the chest and abdomen. A round, 20-mm mass with contrast enhancement was observed in the medial region adjacent to the nipple along with a hyperdense structure thought to be the catheter. (Blue arrow: catheter; red arrow: mass.)

Contrast-enhanced CT of the chest and abdomen: A 20-mm, enhancing mass was seen in the inner quadrant of the left breast and adjacent to the nipple along with a high-density structure presumed to be the catheter (**Fig. 1**). Several, round lymph nodes measuring 12 mm on the short axis were observed in the left axilla. No distant metastases were identified. On chest radiography, abnormal shadows suggestive of catheter calcification were noted in the neck and thoracic sections of the VP shunt pathway.

**Fig. 2:**
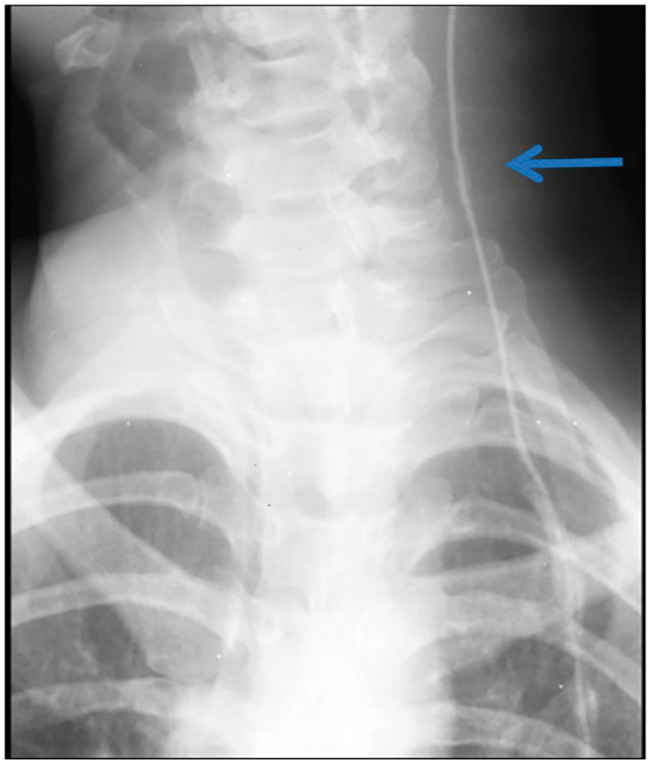
Contrast study of the shunt. The contrast medium smoothly traveled along the catheter into the peritoneal cavity with no evidence of leakage along the catheter or reflux into the ventricular side.

Fluoroscopic shuntography: 5 mL of non-ionic iodinated contrast medium was injected through the retroauricular valve reservoir under continuous fluoroscopic guidance. The opacified column advanced smoothly along the distal catheter into the peritoneal cavity without extravasation or retrograde reflux across the valve and into the ventricular limb (**Fig. 2**). A radiograph obtained 60 minutes later demonstrated complete clearance of the medium, confirming catheter patency. The unidirectional, antegrade flow without retrograde reflux was consistent with a competent valve mechanism.

**Fig. 3:**
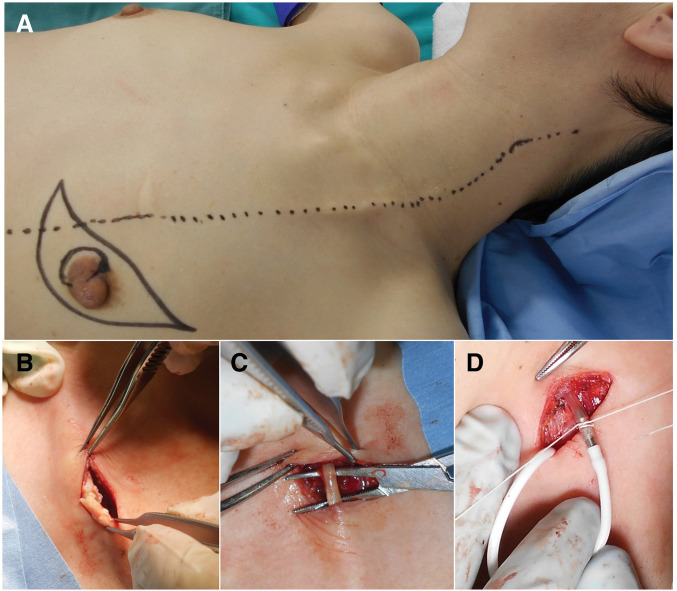
Surgical findings. (**A**) The thoracic course of the VP shunt was marked on the skin via direct visual inspection and ultrasound guidance prior to surgical draping. (**B**) The planned connection site above the clavicle was calcified, and a partial rupture occurred during traction. (**C**) By shifting the skin incision cranially and tracing the catheter upward, an area without calcification, where the catheter’s elasticity was preserved, was identified and chosen as the new connection site. (**D**) The existing and new catheters were connected using an I-shaped titanium connector.

The therapeutic strategy was determined by several considerations. First, the shunt was functional, although its physiological necessity remained uncertain; therefore, preservation of the catheter was deemed desirable whenever feasible. Second, the tumor–catheter distance of 12 mm raised concern regarding the ability to secure adequate oncological margins. Third, the catheter showed extensive calcification, which increased the risk of intraoperative fracture. On the basis of these factors, a staged distal revision was planned immediately prior to mastectomy. The thoracic course of the VP shunt was marked on the skin via direct, visual inspection and ultrasound guidance prior to surgical draping (**Fig. 3A**). Given the extensive catheter calcification observed on CT, distal rerouting of the catheter was planned to achieve a tension-free connection.

A 3-cm skin incision made immediately above the left clavicle exposed the shunt catheter. The preselected connection site was heavily calcified and fractured under minimal traction (**Fig. 3B**). Stepwise cranial dissection identified a 2-cm length of supple, non-calcified tubing that retained its native elasticity; this site was selected for connection (**Fig. 3C**). Once the suitability of the site was confirmed, a new peritoneal catheter (abdominal catheter/823045, CODMAN, Integra Japan, Tokyo, Japan) was inserted through a 1-cm, infra-umbilical incision. The catheter was tunneled subcutaneously across the right supraclavicular fossa and brought to the clavicular wound on the left side, where it was coupled to the preserved proximal segment with an I-shaped titanium connector (titanium connector/823053, CODMAN, Integra Japan) (**Fig. 3D**). Immediately after coupling, clear cerebrospinal fluid (CSF) flowed freely through the catheter, confirming intraluminal patency.

Next, mastectomy and axillary lymph node dissection were performed. The incision and flap formation followed the standard mastectomy approach. The catheter passed through the subcutaneous tissue without penetrating the pectoralis major muscle. However, during excision of the breast tissue, the distal catheter was unable to be removed due to extensive calcification and strong adhesions to the surrounding tissue. Attempts at removal caused it to fracture. The stumps of the peritoneal-side catheter were ligated twice and left *in situ*.

**Fig. 4:**
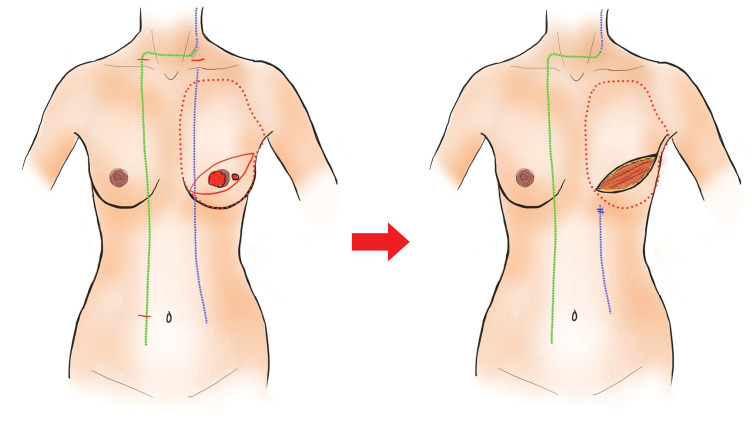
Surgical diagram. The blue line indicates the original catheter, and the green line indicates the catheter after route modification.

**Fig. 5:**
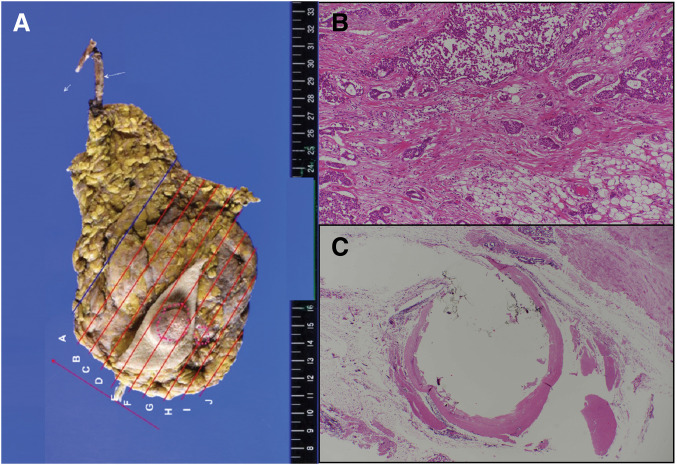
Pathological finding of excised mammary gland. (**A**) The shunt catheter was irregular and had turned white. Accompanying calcification and hyalinization were observed. (**B**) Hematoxylin and eosin staining. Invasive ductal carcinoma with predominantly intraductal components. (**C**) Fibrosis and hyalinization were observed around the catheter along with partial calcification. No evidence of cancer invasion was found.

**Figure 4** shows the intraoperative schematic. Macroscopically, the catheter appeared irregular, had a whitish coloration, and lacked elasticity (**Fig. 5A**).

Pathology:

The two tumors, which measured 22 × 20 × 13 mm and 12 × 10 × 8 mm, were diagnosed as invasive ductal carcinoma, solid type (**Fig. 5B**). The pathologic staging was pT2 (22, 12 mm), pN1(1/11), M0: pStage IIB (TNM, UICC 8th edition). ER (Allred score): proportion score 5 + intensity score 3 = total score 8, PR (Allred score): proportion score 5 + intensity score 3 = total score 8, HER2 score: 1+. There was no evidence of invasion into the shunt catheter. The surrounding tissue exhibited calcification, foreign-body-type granuloma formation, and fibrosis around the catheter (**Fig. 5C**).

The patient’s postoperative recovery was uneventful: no neurological symptoms occurred, and she was discharged on POD 17. Adjuvant hormonal therapy with tamoxifen was administered postoperatively.

At 2 years and 5 months of follow-up, the patient remained asymptomatic and showed no evidence of disease.

## DISCUSSION

The present case involved a VP shunt that had been in place for nearly four decades, resulting in the formation of dense adhesions to the surrounding tissue and progressive catheter calcification. Intraoperative exploration, nevertheless, identified a short, pliable segment that was suitable for reconnection using an I-shaped connector, thus permitting the entire, proximal (ventricular) limb to be preserved.

When breast cancer develops on the same side as a ventriculoperitoneal shunt, three important factors should be considered preoperatively: (1) shunt function and potential dependency; (2) the distance of the lesion from the catheter; and (3) the extent and distribution of catheter calcification. A comprehensive evaluation of these factors is essential to establish the safest and most reliable surgical strategy.

A previous study reported a method of determining whether a functioning shunt should be cut intraoperatively to confirm CSF outflow.^1)^ In our case, contrast medium was injected into the shunt lumen preoperatively to assess the CSF flow through the shunt. Since shuntography visualizes stenosis and ruptures along the entire catheter trajectory, preoperative shunt imaging is considered important. However, the pressure from the injection of the contrast medium can damage a valve which has deteriorated over time. In the present case, the neurosurgeon carefully performed shuntography by injecting approximately 5 mL of contrast medium over several seconds using a 20-mL syringe, thereby avoiding excessive pressure buildup within the shunt lumen.

When immediate reinsertion of the proximal catheter is not feasible, as is often the case in slit ventricles associated with well-controlled hydrocephalus, the shunt may be temporarily ligated, and the intracranial status should be closely monitored before definitive reconstruction.

In this case, surgery was performed in collaboration with neurosurgeons because shunt pathway revision was required. If preserving a shunt is desirable, surgery should be performed with a neurosurgeon present in case the catheter should be damaged.^2)^

A review of the English and Japanese language literature identified 10 previous cases of breast carcinoma associated with a VP shunt; the present case brings the total to 11 (**Table 1**).^1–10)^

**Table 1:** Cases of breast cancer associated with the VP shunt pathway

No.	Ref.	Age	Location	Pathology	Tumor size	Distance between shunt and tumor	Duration of indwelling shunt	Treatment	Shunt	Prognosis/follow-up time
1	4)	52	Rt. CEN	No description	5 cm	Unknown	7 years	Bt	Preserved	Good progress/–
2	1)	68	Lt. UIQ	ILC	3.5 cm	2 cm	11 years	Bt+Ax	Rerouted	–/–
3	5)	88	Rt. UIQ	IDC	1.7 cm	0	Several years	Endocrine therapy	–	–/–
4	6)	70	Rt. UIQ	IDC	8 cm	0	2 years	Bt+Ax	Rerouted	Good progress/2 months
5	7)	67	Rt. UIQ	IDC	1.3 cm	0	7 years	Bp+SNB	Rerouted	Good progress/-
6	8)	52	Rt. LIQ	IDC	2.5 cm	0	4 months	Bt+Ax	Rerouted	Abdominal wall recurrence/14 months
7	9)	74	Rt. CEN, UIQ	IDC	Unknown	Unknown	2 years	Bp	Preserved	Intramammary recurrence/3 years
8	3)	84	Lt. LIQ	No description	3 cm	0	15 years	No treatment	–	Died/10 months
9	2)	62	Lt. CEN, LOQ	IDC (solid-tubular)	5.3 cm	2 cm	44 months	Bt+SNB, RT	Preserved	Good progress/29 months
10	10)	86	Lt. 9 o’clock	IDC	3.6 cm	0	Unknown	Bt	Rerouted	Good progress/4 months
11	Ours	46	Lt. CEN, 3 o’clock	IDC	20 cm	1.2 cm	About 40 years	Bt+Ax	Rerouted	Good progress/29 months

A search of both Japanese and English language case reports of breast cancer in patients with a ventriculoperitoneal (VP) shunt yielded 10 cases. The present case brought the total number to 11.

Ax, axillary lymph node dissection; Bp, breast partial mastectomy; Bt, breast total mastectomy; CEN, central portion; IDC, invasive ductal carcinoma; ILC, invasive lobular carcinoma; LIQ, lower inner quadrant; LOQ, lower outer quadrant; Lt., left; NIP, nipple; Rt., right; RT, radiation therapy; SNB, sentinel lymph node biopsy; UIQ, upper inner quadrant; VP shunt, ventriculoperitoneal shunt

The decision of whether to perform mastectomy or breast-conserving surgery may also influence the surgical approach. Due to the small number of previous reports, no consensus has yet been reached on this matter. In the present case, the tumor lay only 12 mm from the calcified shunt tract, and adjuvant radiotherapy would have been technically difficult; therefore, total mastectomy and shunt rerouting were chosen.

However, since there was no cancer infiltration around the catheter, catheter-preserving total mastectomy may have been an option, while the consultation with a neurosurgeon was indispensable.

Calcification is reportedly more common in young patients and in shunts that have been in place for over 5 years.^11)^ In the present case, approximately 40 years had passed since catheter implantation, one of the longest durations hitherto reported. The patient had severe intellectual disability and had refused hospital visits for 40 years, making follow-up impossible during that period. Fortunately, the heavily calcified portion of the catheter was part of the segment which was removed during shunt rerouting, thus eliminating the need to replace the segment closest to the clavicle. However, preoperative X-rays and CT studies underestimated the extent of calcification, highlighting the difficulty of precisely ascertaining the state of calcification.

Preoperative collaboration with neurosurgeons to perform a thorough evaluation and prepare for surgery is essential for anticipating issues related to a VP shunt in a case such as ours.

Finally, lifelong neurosurgical follow-up remains essential: catheter segments not revised at the index surgery may deteriorate with time, and careful surveillance is required to complement routine cancer care.

## CONCLUSIONS

In breast cancer surgery performed ipsilaterally to a shunt catheter, the necessity of revising the shunt pathway depends on factors such as the distance from the shunt function, condition of the catheter, and histological type of the tumor. However, as of yet there is no consensus on the optimal management of this complex situation. Moreover, when a shunt catheter has been in place for an extended period, the potential effects of calcification must be taken into account. It is essential to prepare for issues, such as catheter damage, or scenarios in which revising the shunt route may not be feasible.

In a situation such as those described above, the risk of hydrocephalus recurrence should be preoperatively assessed in collaboration with neurosurgeons to ensure that the surgery is carefully planned and prepared.

## DECLARATIONS

### Availability of data and materials

The datasets used and/or analyzed during the current study are available from the corresponding author upon reasonable request.
